# The Multifaceted Role of WNT Signaling in Alzheimer’s Disease Onset and Age-Related Progression

**DOI:** 10.3390/cells12081204

**Published:** 2023-04-21

**Authors:** William W. Kostes, David A. Brafman

**Affiliations:** School of Biological and Health Systems Engineering, Arizona State University, Tempe, AZ 85287, USA

**Keywords:** WNT, Alzheimer’s disease, central nervous system, amyloid, tau, inflammation, synaptic function

## Abstract

The evolutionary conserved WNT signaling pathway orchestrates numerous complex biological processes during development and is critical to the maintenance of tissue integrity and homeostasis in the adult. As it relates to the central nervous system, WNT signaling plays several roles as it relates to neurogenesis, synaptic formation, memory, and learning. Thus, dysfunction of this pathway is associated with multiple diseases and disorders, including several neurodegenerative disorders. Alzheimer’s disease (AD) is characterized by several pathologies, synaptic dysfunction, and cognitive decline. In this review, we will discuss the various epidemiological, clinical, and animal studies that demonstrate a precise link between aberrant WNT signaling and AD-associated pathologies. In turn, we will discuss the manner in which WNT signaling influences multiple molecular, biochemical, and cellular pathways upstream of these end-point pathologies. Finally, we will discuss how merging tools and technologies can be used to generate next generation cellular models to dissect the relationship between WNT signaling and AD.

## 1. Introduction

Alzheimer’s disease (AD) affects over six million individuals in the U.S. and has a direct cost estimated in excess of USD 400 billion/year [1]. Broadly speaking, there are two forms of AD—early-onset, familial AD (FAD) and late-onset-sporadic AD (SAD). Numerous molecular signaling pathways have been implicated in both FAD and SAD. In particular, aberrant WNT signaling, an evolutionary conserved pathway that orchestrates numerous biological processes, has been implicated in the manifestation and propagation of AD-related phenotypes. Thus, a more thorough understanding of how dysfunction in WNT signaling leads to onset and age-related progression of AD will lead to the development of therapeutic interventions. 

To that end, this review will first detail the molecular mechanisms and key pathological features of AD. We will also provide an overview of the WNT signaling pathway. Next, we will discuss the in vitro, in vivo, and clinical evidence that exists for a relationship between AD and the WNT pathway. In turn, we will describe the various potential mechanisms by which the WNT pathway modulates AD-related molecular and cellular pathologies. Finally, we discuss emerging tools and technologies that can be employed to further dissect the mechanisms by which WNT signaling induces AD-associated pathologies and cognitive dysfunction.

## 2. Alzheimer’s Disease

Classical neuropathological hallmarks of AD include extracellular aggregation of amyloid plaques and neurofibrillary tangles formed by the aggregation of hyperphosphorylated tau [2]. Additional pathologies such as cortical thinning and atrophy attributed to synaptic and neuronal loss have been observed throughout the disease progression [2]. In addition to these pathological features, post-mortem analysis of AD individuals reveal evidence of cerebral angiopathy and systemic brain inflammation [3]. However, the hierarchy of these pathologies and how they result in cognitive decline in AD patients remains uncertain and highly debated [4]. In this section, we will highlight the well-established pathological mechanisms and features of AD.

### 2.1. Amyloid-β (Aβ) Induced Pathologies in AD

In the strictest form of the amyloid cascade hypothesis, generation, and subsequent oligomerization of Aβ is the key step that leads to elevated phosphorylated-tau, inflammation, and subsequent neuronal loss. In the central nervous system, neurons act as the primary producers of Aβ from the amyloidogenic processing of amyloid precursor protein (APP) whereas non-neuronal cells, such as astrocytes and microglia, facilitate its clearance [5]. The imbalance in these processes is thought to be a primary driver of elevated extracellular Aβ levels found in AD patients [6,7]. Extracellular Aβ takes on different structures as accumulation increases, beginning first as Aβ oligomers and progressing as fibrils and lastly senile plaques. It has been speculated that these different forms may contribute to neurodegeneration during different stages of AD [8]. The aggregation of Aβ has been shown to produce toxins and reactive oxygen species which disrupt neuronal membranes by impairing the function of ion-motive ATPases, glucose transporters, and glutamate transporters disrupting neuronal membranes and ultimately resulting in dysfunction of normal cell physiological processes and eventual synaptic and neuronal loss [9,10,11].

Once synthesized, APP is transported through the secretory pathway by moving from the endoplasmic reticulum (ER) through the Golgi complex and trans-Golgi network to the plasma membrane [12,13,14]. During transit, APP can be subject to extensive post-translational modifications, including phosphorylation [15]. At the plasma membrane, APP can be processed in the non-amyloidogenic pathway through cleavage by α-secretase to release soluble sAPPα. The remaining α-C-terminal fragment (α-CTF) is further processed by γ-secretase which produces the p3 peptide and the APP intracellular domain (AICD). Alternatively, APP not processed in the non-amyloidogenic pathway is internalized from the cell surface through clathrin-mediated endocytosis and enters early endosomes [14]. From early endosomes, a subset of APP molecules can undergo recycling to the cell surface [16,17]. Alternatively, maturation of early endosomes into late endosomes where the acidic environment elicits β-secretase cleavage generates soluble sAPPβ and the β-C-terminal fragment (β-CTF). Subsequent cleavage by γ-secretase leads to the generation of Aβ. The resultant Aβ species are either targeted for degradation in the lysosome or release into the extracellular space via exocytosis. Several proteolytic enzymes function to degrade Aβ including neprilysin, endothelin converting enzymes 1 and 2, insulin-degrading enzyme, and plasmin [18]. Extracellular Aβ can be found in many different forms in the brain including monomers that can form structures such as oligomers, protofibrils, and amyloid fibrils [18]. Oligomers are a soluble form of Aβ found throughout the brain and amyloid fibrils are large and insoluble structures that later form amyloid plaques.

In AD patient brains, the various cleaved products of APP amyloidogenic processing accumulate inside neurons [19]. In particular, increased intraneuronal accumulation of CTF-β in enlarged endosomal compartments is a pathological hallmark of AD progression and thought to contribute, in part, to downstream synaptic loss [17]. In addition, it has been speculated that impaired lysosomal function could cause a shift from intraneuronal Aβ degradation to an increase in extracellular Aβ release [20,21,22]. In particular, it has been suggested in the context of FAD-related mutations that reduced lysosomal degradation of proteins is a consequence of alterations in lysosomal pH [23,24]. Along similar lines, dysfunction of proteolytic enzymes associated with degradation of Aβ were found to cause a deposition of Aβ plaques in the cerebrum [25].

Astrocytes and microglia play a critical role as it relates to extracellular Aβ uptake and degradation [26]. Aβ can be endocytosed by astrocytes through indirect or direct association with APOE in the context of several receptors mainly LDLR, LRP1, and HSPG [27,28,29]. With respect to microglia, it has been shown that TREM2 binds to APOE to facilitate uptake of Aβ [30]. After receptor-mediated endocytosis, Aβ traffics through the early and late endosomes to the lysosomes for degradation [31,32]. In addition to uptake by astrocytes and microglia, Aβ can be cleared from the extracellular space through transport across the brain to the blood–brain barrier (BBB). The exchange of Aβ across the BBB is regulated by the receptors RAGE [33] and LRP1 [33,34].

As it relates to AD, recent studies have suggested that the amyloid pathologies that are more commonly associated with genetic risk factors might be due to endocytic defects associated with Aβ uptake in glia cells [35,36]. Along similar lines, impairment of Aβ endosomal trafficking to the lysosomes can lead to defective Aβ uptake [31,37,38]. In particular, defective lysosomal degradation observed in older adults and FAD patients has been implicated in contributing to AD pathogenesis [39,40,41,42,43]. In addition, it has been demonstrated that defective clearance of Aβ across the BBB may contribute to the buildup of neurotoxic Aβ associated with AD [44].

### 2.2. Tau-Related Pathways in AD

Tau is an unfolded hydrophilic protein largely present in the axons of developing neurons and encoded by the *MAPT* gene [45,46,47,48]. In vitro studies have shown that tau functions to promote microtubule assembly and maintain stability [49]. However, in vivo studies have revealed opposing functions as knockout or knockdown of tau did not show impaired microtubule assembly or axonal transport in primary neurons [50,51,52]. Studies using tau knockout mice suggest that overt phenotypes are not observed due to functional compensation by other microtubule associated proteins such as MAP1A, MAP1B, and MAP2 [53,54,55]. This finding is further supported by evidence from humans developing normally despite disease-causing mutations that result in loss or aberrant production of tau [56]. 

In healthy neurons, tau functions in synaptic activity in the postsynaptic compartment where it associates with microtubules in axons, as well as dendrites at the post-synapse under pathological conditions [57,58]. Studies have revealed that tau plays a role in maintaining neuronal projections that affect synaptic function by acting as a promoter of axonal microtubule assembly [59,60,61]. In turn, synaptic function may be impaired due to loss of tau binding to microtubules. In vivo investigations of tau-deficient mice showed impairment of synaptic potentials and defects in spatial reversal learning [62,63]. Another study discovered that tau knockdown in the adult hippocampus impaired motor coordination and caused morphological synaptic defects [64]. 

Neurofibrillary tangles (NFTs) consisting of hyperphosphorylated tau are a pathological hallmark of AD [65]. In AD brains, hyperphosphorylated tau levels are three- to four-fold higher compared to a normal adult brain [66]. Tau function is disrupted by phosphorylation as it is no longer able to bind to microtubules [67,68,69,70]. Furthermore, phosphorylation of tau induces tau self-assembly into tangles and filaments, precursors to NFTs and AD pathogenesis [71]. In addition, hyperphosphorylated dendritic tau [72,73,74] has been shown to be a key driver in dendritic loss, aberrant postsynaptic activity, and cognitive dysfunction in AD [75,76]. At the pre-synapse, hyperphosphorylated tau has been observed to disrupt normal synaptic vesicle release as it forms more stable interactions with synaptic vesicles seen in AD brains that are absent in control brains [77,78,79,80].

More recently, it has been suggested that tau pathology in AD is mediated, in part, by the release, uptake, and prion-like spread of pathogenic tau aggregates between synaptically connected neurons [81,82,83,84,85]. Although endo-lysosomal dysfunction can indirectly lead to tau pathologies due to elevated amyloid levels, dysfunction of endocytosis might modulate not only tau phosphorylation and aggregation [86,87,88] but also internalization of tau [89,90,91]. Specifically, recent work has suggested that pathological tau can be released, in part, through the action of exosomes [92,93] and internalized by other neurons through receptor mediated endocytosis [85,91,94]. 

Numerous studies support an Aβ-dependent interaction with tau protein during the progression of AD. The presence of Aβ has been shown to enhance tau pathology and accumulation throughout the stages of AD [95,96], as well as accelerate the formation of high molecular weight hyperphosphorylated tau [97]. In addition, patients possessing both Aβ-plaques and pathological tau displayed greater levels of high molecular weight hyperphosphorylated tau compared to individuals with pathological tau and negligible Aβ levels [95,98]. Analysis of a transgenic mouse model possessing both Aβ and tau pathology revealed that Aβ plaques developed prior to tau tangles and that Aβ antibodies alleviated early-disease tau modifications [99]. Along similar lines, overexpression of the genes *APP* and *PSEN1* (with wild-type tau) induced Aβ and tau aggregation, causing tau pathology [100]. The use of 𝛽- or 𝛾-secretase inhibitors blocked Aβ production and prevented associated tau pathology in neural cells from human induced pluripotent stem cells (hiPSCs) of AD patients, suggesting aberrant APP processing contributes to Aβ and tau formation [101,102]. 

However, emerging evidence from in vitro and in vivo studies suggests that tau pathology can arise independently from Aβ accumulation. For example, several studies have suggested that amyloid-independent induction of tau pathologies can arise from dysfunction in the endocytic system [103]. In fact, early studies have suggested that modulation of genes associated with the endo-lysosomal pathway can induce tau-related phenotypes in an amyloid-independent manner [103,104]. For instance, studies have shown that the endocytosis-related proteins BIN1 and PICALM can regulate tau pathology independent of Aβ pathology [104]. Although pathological tau generation mainly occurs in neurons, prorogation of tau not only occurs in neurons but also astrocytes and microglia [105,106]. However, unlike neurons, astrocytes and microglia also have the ability to degrade pathological tau, which might explain the cell-specific tau pathologies observed in AD associated with defective endo-lysosomal transport [105,106]. Cholesterol metabolism has also been shown to be an amyloid-independent regulator of tau pathology. AD brains have shown abnormal accumulation of cholesteryl esters [107] which have been observed to inhibit the proteosomal degradation of tau [108] resulting in increased deposition of phosphorylated tau [108,109]. In a mouse model, inhibition of cholesterol synthesis reduced tau pathology in tau transgenic mice despite the absence of Aβ pathology, suggesting tau pathology independent of Aβ [110].

### 2.3. Inflammatory Processes in AD

Several inflammatory processes are identifiable at various stages of AD including glia activation, microgliosis, astrogliosis, and cytokine release [111,112,113]. Although it is generally thought that these inflammatory processes act downstream of Aβ and tau, recent evidence suggests glia cell activation and increased expression of inflammatory-related cytokines may exacerbate both amyloid and tau pathologies [114]. 

With regards to Aβ-related pathologies gliosis and neuroinflammation have been shown to function as a double-edged sword during AD, either offering protective or damaging effects depending on cell type [115]. For example, reactive microglia and astrocytes can clear Aβ, critical for mitigating Aβ deposition [116]. On the other hand, Aβ aggregation can cause inflammatory responses and release of inflammation associated mediators such as eicosanoids, chemokines, pro-inflammatory cytokines, and complement factors. Following this inflammatory response, increased neuronal death and loss of neuronal synapses occur. Consequently, clearance of Aβ and neuronal debris mediated by microglia are impaired. Studies have also shown that soluble Aβ can elicit neuronal dysfunction by stimulating pro-inflammatory activation of primary microglia [117]. In turn, sustained levels of Aβ can trigger a response by the immune system via microglial activation and subsequent neuronal loss by phagocytosis [118]. In addition, microglial activation can induce production of pro-inflammatory cytokines that cause neuronal death [119,120]. Mechanistically, numerous microglia receptors are associated with Aβ clearance and stimulating inflammatory responses. The receptors SR-AI and TREM2 are responsible for the clearance of Aβ by internalization of Aβ fibrils whereas other receptors RAGE and NLRP3 are implicated in triggering a pro-inflammatory response that activates the production of pro-inflammatory mediators [100,121]. Complement receptors, Fc receptors, FPRL1/FPR2, CD36, and TLRs are involved in both Aβ clearance and initiating an inflammatory response. 

In relation to tau-related neuroinflammatory processes, a growing number of studies are providing evidence that neuroinflammation precedes tau misfolding and neurodegeneration. In a humanized mouse model, microglia activation caused neuronal loss and impaired synaptic function prior to the formation of tau fibrillary tangles [122]. In another study, an increased production of cytokines including TNF-𝛼, IL-1β, and GM-CSF were detected in the CSF of patients displaying mild cognitive impairment, suggestive of an inflammatory response prior to full expression of definitive tau pathology [123]. Other studies have revealed activated microglia secrete TNF-𝛼 and IL-1β which act to promote tau hyperphosphorylation and aggregation by CDK5 and p38 MAPK signaling [124,125,126,127]. Along similar lines, the microglia receptor NLRP3 in activated microglia has been shown to drive aberrant tau phosphorylation and misfolding via CAMKII [128]. 

### 2.4. Synaptic Loss in AD

Analysis of postmortem brain samples of AD patients has revealed a strong correlation between synaptic loss and AD-associated pathologies [129,130,131,132,133,134]. Studies using human FAD mouse models corroborated this correlation, having observed synaptic dysfunction including impaired plasticity and synapse loss [59,135,136,137,138,139]. 

Mechanistically, synaptic loss arises from other AD-associated pathologies including Aβ aggregation, tau hyperphosphorylation, and activated microglia. The aggregation of Aβ has several deleterious effects on synaptic function including impaired synaptic activity and increased synapse loss. Both presynaptic [140,141] and postsynaptic [142] activity have been shown to modulate the release of Aβ from neurons, suggesting a synaptic regulatory role of extracellular Aβ. With respect to tau pathology, hyperphosphorylated tau impaired synaptic function by inhibiting either glutamate receptor trafficking or synaptic anchoring [76]. In addition, the spreading of tau aggregates has been associated with subsequent synaptic dysfunction, synapse loss, and neurodegeneration [143]. In regards to inflammatory processes and synaptic health, one study demonstrated that microglia contribute to synaptic loss through Aβ-induced microglia phagocytosis of synaptic contents [144]. In addition, during this process, it has been shown that activated microglia employ inflammatory cytokines which can induce synaptic dysfunction [145,146,147]. 

## 3. WNT Signaling 

**Figure 1:**
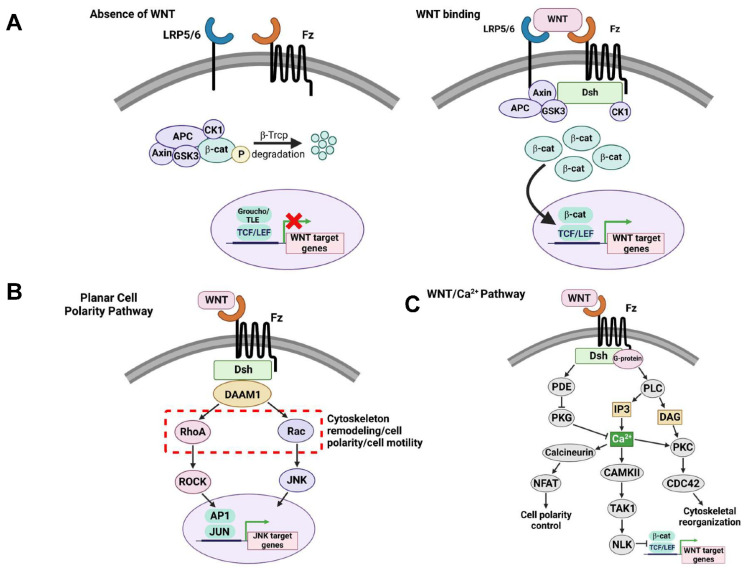
**WNT signaling pathways**. (**A**) Canonical WNT/𝛽-catenin signaling pathway. Absence of WNT ligand: WNT signaling is in an “off” state when a WNT ligand does not bind to the receptors Frizzled (Fz) and LRP5/6. In turn, the destruction complex (APC, Axin, CK1, GSK-3𝛽) will phosphorylate cytoplasmic 𝛽-catenin leading to proteosomal degradation. WNT binding: WNT signaling is turned “on” when a WNT ligand binds to the receptors Fz and LRP5/6. Following binding, the Disheveled (Dsh) scaffolding protein recruits and complexes with the destruction complex, subsequently impairing 𝛽-catenin phosphorylation and degradation. 𝛽-catenin will accumulate in the cytoplasm where it will translocate to the nucleus and interact with TCF/LEF factors to regulate transcription of WNT target genes. (**B**) Non-canonical WNT/Planar Cell Polarity (PCP) signaling pathway. Activation of WNT/PCP is independent of 𝛽-catenin and does not require WNT ligand binding to LRP5/6 receptor. After WNT ligand binding, Dsh is activated which then activates RhoA and Rac1, resulting in activation of ROCK and JNK signaling, respectively. Activation of these signaling pathways mediate cytoskeleton remodeling, cell polarity, and cell motility. (**C**) Non-canonical WNT/Ca^2+^ signaling pathway. WNT ligands bind to Fz inducing the activation and recruitment of Dsh and G-proteins. Here, Dsh activates phosphodiesterase PDE which inhibits PKG and subsequent release of calcium ions. In addition, Dsh activation induces expression of PLC which functions to activate IP3 and DAG. IP3 promotes the release of calcium ions which activates expression of: Calcineurin and subsequently NFAT leading to greater control of cell polarity, CAMKII which induces TAK1 and NLK to repress WNT target gene transcription, and lastly, PKC which promotes CDC42 expression and downstream cytoskeletal rearrangement.

The WNT signaling pathway is comprised of 19 ligands and 10 Frizzled (Fzd) receptors. Binding of these secreted lipoproteins to extracellular receptors leads to activation of WNT signaling and regulation of other signaling pathways. The WNT signaling pathway has been characterized into three separate pathways—canonical WNT/β-catenin signaling, planar cell polarity (PCP), and WNT/Ca^2+^ (Figure 1). The canonical WNT/β-catenin pathway is described by WNT ligands binding to a Fzd receptor and LRP5/6 co-receptor. In turn, this leads to the disassembly of the destruction complex and inhibits ubiquitylation of cytoplasmic β-catenin by GSK3β [148]. β-catenin is then allowed to accumulate in the cytoplasm and translocate to the nucleus where it interacts and binds with T-cell factor/lymphoid enhancer factors (TCF/LEF) to regulate WNT target gene expression [149]. Several factors have been identified which negatively regulate WNT signaling including WIF-1, SFRP1, SFRP3, SFRP4, DKK1, and DKK3 [150]. WIF-1 functions to negatively regulate WNT via inducing PI3K/Akt/mTOR signaling which leads to reduced expression of Dvl2 and downstream downregulation of 𝛽-catenin [151]. In general, SFRPs inhibit WNT signaling by binding WNT ligands, preventing interactions with Fzd to activate WNT signaling. Both SFRP1 and SFRP3 inhibit 𝛽-catenin transcription [152,153], however SFRP1 also decreases levels of phosphorylated GSK3𝛽 which cause canonical WNT inhibition [152]. SFRP3 functions to downregulate WNT signaling by binding to WNT1 to inhibit interactions between WNT1 and Fzd receptor [153]. SFRP4 has been shown to also inhibit WNT signaling by decreasing 𝛽-catenin [154]. Other WNT inhibitors include DKK1 and DKK3. DKK1 inhibits WNT by binding to the LRP5/6 co-receptor, leading to increased 𝛽-catenin degradation [155]. DKK3 acts as an inhibitor of WNT by decreasing both 𝛽-catenin expression and TCF4 activity [156]. The WNT PCP pathway is activated by WNT ligands exclusively binding to the Fzd receptor, without the need of LRP5/6 co-receptors [157]. As a result, this causes activation of the small GTPases RhoA and Rac1, which then activate kinases ROCK and JNK, respectively [157]. The WNT/PCP pathway is responsible for transcriptional changes that direct cytoskeleton reorganization. The WNT/Ca^2+^ signaling pathway features the binding of WNT ligands to Fzd receptors, which in turn leads to the activation of phospholipase C (PLC) activation causing release of Ca^2+^ from intracellular stores and activation of CaMKII and PKC, consequently causing transcriptional changes and actin remodeling [158]. Fzd and LRP5/6 are not exclusive receptors of WNT ligands as other receptors have been identified such as receptor tyrosine kinase like orphan receptors 1 and 2 (ROR1 and 2), as well as the receptor-like tyrosine kinase RYK [159]. Interestingly, of the two receptors, ROR2 is the only one to demonstrate tyrosine kinase activity [160], whereas ROR1 and RYK are speculated to function as pseudokinases. WNT ligands interact with the cysteine rich domain of ROR1 and 2 similarly to that of Fzd. The RYK receptor harbors an extracellular WNT inhibitory factor (WIF) domain that directly binds WNT [161]. As it relates to AD, following knockdown of either ROR1 or 2, a significant decrease in synapse formation was observed [162]. In the same study, these receptors were discovered to interact with WNT5a to mediate presynaptic clustering, suggesting a key role of these receptors in WNT5a-mediated synapse formation. In this section, we will discuss the role of WNT signaling in neurogenesis, synaptic plasticity, memory and learning.

### 3.1. The Role of WNT Signaling in Neurogenesis

Adult neurogenesis can be characterized by the generation of mature neurons from adult neural stem cells [163]. Composed of blood vessels, astrocytes, ependymal cells, and mature neurons, the neurogenic niche maintains the development of neural stem cells (NSC) [163]. The canonical WNT/β-catenin pathway has been identified as a regulator of adult hippocampal neurogenesis evidenced by active WNT signaling in the hippocampal neurogenic niche from astrocytes [164,165,166]. Studies have revealed secreted WNT molecules as key for normal brain development including postnatal neurogenesis by regulating NSC self-renewal, proliferation, and differentiation [164,167]. More specifically, Wnt3 production by astrocytes was shown to differentiate adult NSCs [165]. Another study demonstrated that overexpression of Wnt3 increased neurogenesis from adult hippocampal stem/progenitor cells (AHPs) and that WNT inhibition reduced neurogenesis of AHPs in vitro and nearly prevented all neurogenesis in vivo [164]. In addition, the ligand Wnt7a has been shown to be essential for NSC self-renewal, neural progenitor cell cycle progression, and neuronal differentiation and maturation [168]. The loss of Wnt7a led to a decrease in the number of newborn neurons in the adult hippocampal dentate gyrus [168]. In addition, Wnt7a stimulated neural stem cell proliferation by activating β-catenin D1 pathway and promoted neuronal differentiation and maturation by inducing β-catenin neurogenin 2 pathway [168]. 

Studies have shown a decrease in neurogenesis with aging, attributing changes in the neurogenic niche [169,170]. It is speculated that age-related decline is a cause of either a deficit in proliferation or differentiation of potential NSCs. In this regard, Wnt3 has shown to play a role in the decline in neurogenesis [165]. Specifically, Wnt3 production by astrocytes decreased with aging and correlated to decreased neurogenic differentiation of NPCs in an aged brain leading to impairment of neurogenesis [164,171]. 

### 3.2. WNT Signaling in Synaptic Health and Plasticity

WNT signaling has been shown to play a role in regulating synaptic plasticity, as well as modulate neuronal firing [172,173]. Interestingly, WNT was discovered to function in a positive feedback loop in which WNTs change the spiking properties of cells resulting in structural changes that release WNT ligands [174]. In this vein, active signaling of both the canonical WNT/β-catenin and noncanonical WNT/Ca^2+^ pathways were shown to be crucial to regulating the firing activity of neurons in the hippocampus. In the entorhinal-hippocampus circuit, expression of canonical Wnt3a disrupted neural oscillation, whereas expression on noncanonical Wnt5a ligand increased oscillation in new cells [174]. Along similar lines, in hippocampal neurons, Wnt5a activates the non-canonical WNT/Ca^2+^ pathway to modulate dendritic spine morphogenesis and synaptic function [175]. 

It also has been demonstrated that WNT secreted proteins regulate synapse development and are crucial for the early stages of long-term potentiation, a form of plasticity that increases synapse strength [172,173]. Inhibition of WNT via sFRPs revealed that long-term potentiation was impaired, while the addition of exogenous WNT proteins facilitated long-term potentiation [176]. Other studies that inhibited WNT signaling with sFRPs found reduced glutamatergic neurotransmission demonstrating WNT as an important factor for synaptic transmission at mature synapses [175,177,178,179]. In support of this role, the addition of exogenous WNTs was shown to enhance excitatory synaptic transmission in hippocampal neurons [175,178,180,181,182,183]. In particular, Wnt7a was found to stimulate excitatory synapse formation and function [176,183]. 

### 3.3. WNT Signaling in Memory and Learning

Through its effects in neurogenesis and synaptic function, WNT signaling has been shown to play critical roles in memory consolidation and learning. For example, spatial learning results in activated canonical WNT signaling and increased the expression of Wnt5a and Wnt7a/b [184]. Similarly, an investigation of hippocampal memory consolidation in mice revealed that WNT signaling was necessary for object recognition memory consolidation [185]. Alternatively, WNT inhibition with Dkk1 blocked fear learning in mice [186]. In this same study, Dkk1 was shown to impair hippocampal object recognition memory by rapidly reducing the levels of WNT-associated proteins including: β-catenin, cyclin D1, c-myc, Wnt7a, and PSD95 [185]. Together, these findings suggest that learning activates WNT signaling necessary for hippocampal memory consolidation. 

Further studies have suggested that WNT signaling selectively functions in long-term memory and learning [184]. For example, following water maze experiment in rats, elevated levels of Wnt7 and Wnt5a were detected 7 and 30 days after training in granule cells of the hippocampus, but not in CA3 neurons [184]. Moreover, Tabatadze and colleagues observed that both canonical and non-canonical WNT signaling regulated long-term information storage in a behavioral-, cellular-, and isoform-specific manner. 

## 4. WNT Signaling and Alzheimer’s Disease

**Figure 2:**
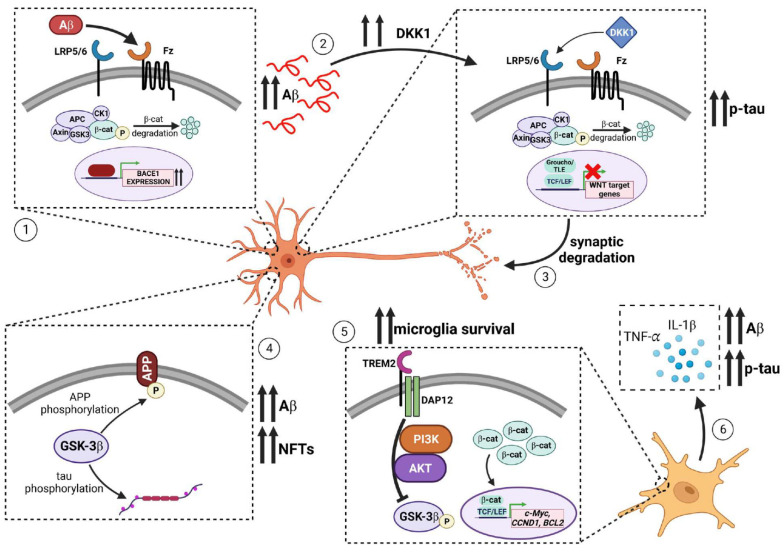
**Effects of WNT on different AD-related pathologies with specific mechanisms.** (**1**) A𝛽 binds to Fz subsequently inhibiting WNT signaling. During WNT inhibition, TCF4 is unable to complex with 𝛽-catenin and repress *BACE1* expression. As a result, BACE1 promotes amyloidogenic processing of APP and increased accumulation of A𝛽. (**2**) Increased deposition of A𝛽 induces expression of the WNT antagonist DKK1. DKK1 binds to LRP5/6, inhibiting canonical WNT/𝛽-catenin signaling and downstream repression of WNT target genes. In turn, phosphorylated tau levels are increased in addition to associated A𝛽-neurotoxicity. (**3**) A𝛽-mediated expression of DKK1 also causes synaptic degradation through the disassembly of synaptic components. (**4**) GSK3𝛽 promotes amyloidogenic processing of APP via phosphorylation resulting in greater levels of A𝛽. Additionally, GSK3𝛽 can phosphorylate tau which leads to the formation of neurofibrillary tangles (NFTs). (**5**) TREM2 stabilizes 𝛽-catenin by interacting with DAP12 to activate PI3K/Akt signaling which phosphorylates and inhibits GSK3𝛽 activity. 𝛽-catenin then translocates to the nucleus to regulate genes that promote microglia survival including *c-Myc*, *CCND1*, and *BCL2*. (**6**) Loss of WNT receptor LRP6 led to a significant increase in microglia activation and neuroinflammation. Activated microglia promote tau hyperphosphorylation and A𝛽 deposition through the release of IL-1β and TNF-𝛼, pro-inflammatory cytokines. Figure 2 was generated with the assistance of Biorender.

Because of the role of WNT signaling in numerous processes related to neural health and homeostasis, the role of aberrant WNT signaling in the onset and age-related progression of AD has been significantly studied. More recently, WNT has been implicated in the dysfunction of the blood–brain barrier (BBB) in AD. Briefly, studies have examined the effects of WNT and the BBB in AD revealing a correlation between WNT dysregulation and BBB breakdown contributing to AD-associated phenotypes [187,188,189]. In this review, we will focus on WNT and neural cell types, however we encourage readers to reference these studies for a greater examination between WNT and BBB during AD. In this section, we will review the genetic, clinical, and animal-based studies that demonstrated the relationship between WNT signaling and AD. In addition, we will discuss the biochemical, molecular, and cellular mechanisms by which WNT signaling influences AD-related pathologies. An overview of these potential mechanisms is provided in Figure 2. In addition, a summary of the role of canonical versus non-canonical WNT signaling pathways in modulating AD-related phenotypes is provided in Table 1. 

### 4.1. Evidence for the Role of WNT Signaling in Alzheimer’s Disease

Several epidemiological, clinical, and animal-based studies have established the relationship between WNT signaling and AD-associated phenotypes. To that end, several studies have shown that GSK3β activity is increased whereas β-catenin levels are decreased in AD patient brains [209,210,211,212]. Moreover, it has been suggested that WNT signaling is impaired in the cortical regions of AD patients through the action of DKK1, a negative modulator of WNT signaling [213]. Other studies of the aging human brain revealed the expression of several WNT ligands including *WNT2b*, *WNT6*, *WNT7a* and frizzled receptors *FZD2* and *FZD3* are downregulated [214]. In the same study, the WNT antagonist *SFRP1* was found to be upregulated. Collectively, increased expression of *SFRP1* could explain the downregulation of WNT ligands in the aging human brain, suggesting a protective effect of WNT against AD-related processes. Interestingly, long-term activation of WNT signaling through the administration of lithium has been shown to induce neuroprotective effects that may slow the cognitive and functional deficits associated with AD [215]. As such, numerous preclinical and clinical studies are investigating a paradigm in which WNT signaling is activated through the use of GSK3β to treat AD [216].

In support of these clinical findings, animal models have been employed to identify potential mechanistic links between WNT signaling and the development of AD. In general, these studies have shown dysregulation of WNT signaling as a common factor contributing to age-associated pathologies in the rodent brain. Downregulation of several WNT ligands such as *Wnt2*, *Wnt3*, *Wnt3a*, *Wnt4*, and *Wnt9a*, as well as the transcription factors *Lef1* and *Tcf3* was observed in aging rodent brains [171,217]. Similar studies in both aging rats and mice found reduced levels of Dvl2, Axin2, and nuclear β-catenin in the hippocampus, suggesting a dampening of WNT signal [218,219]. Reduced levels of WNT signaling in aging animals have also been attributed to increased expression of the WNT antagonist *Dkk1* expressed in rodent hippocampus [220,221]. As it relates to AD-associated pathologies, loss of canonical WNT/β-catenin signaling has been shown to induce AD-like neuropathological hallmarks in wild-type mice, and accelerate the development of AD-like pathology in an AD mouse model [222]. Along similar lines, analysis of a mouse model with an inducible *Dkk1* expression cassette revealed that overexpression of *Dkk1* in the adult brain reproduces several phenotypes in AD mouse models such as reduced synaptic transmission, impaired long-term potentiation, enhanced long-term depression, and long-term memory loss [223,224]. 

Overall, from these studies, a general model has emerged in which downregulation of WNT signaling occurs in AD whereas activation of WNT signaling may be protective against induction of AD-related phenotypes. 

### 4.2. Amyloid-Centric Mechanisms by which WNT Modulates AD-Related Phenotypes

#### 4.2.1. WNT and APP Post-Translational Modification

After generation in the ER, APP undergoes post-translational modifications and reaches the cell surface through the constitutive secretory pathway [225]. In particular, phosphorylation of APP at T688 has been extensively studied because of its association with AD as well as its effect on amyloidogenic processing of APP [225]. A variety of kinases are capable of phosphorylating the T668 residue including ERK1/2, GSK3β, CDK5, and JNK [190,191,226,227]. GSK3β activity is inhibited during active canonical WNT/β-catenin signaling, whereas JNK is expressed during active WNT/PCP signaling. Together, this suggests elucidating the mechanisms of WNT signaling and its effects on APP processing will require a nuanced approach in differentiating between canonical and non-canonical WNT pathways. 

Previous studies in neurons support these findings, demonstrating that APP is mainly phosphorylated by GSK3β and JNK kinases [228,229]. In particular, several lines of evidence suggest that GSK3β modulates APP processing resulting in increased production of Aβ [230]. For example, GSK3β phosphorylation of APP has been shown to modulate amyloidogenic and non-amyloidogenic APP processing at the T668 amino acid site of APP [15,225]. In addition, JNK activation enhanced APP phosphorylation at the same amino acid site, favoring amyloidogenic cleavage of APP. The same study also showed inhibition of the JNK pathway reduced Aβ deposition in neurons by shifting APP processing to the non-amyloidogenic pathway [231]. 

#### 4.2.2. WNT Signaling and APP Endocytosis

The levels of cell surface APP and rate of endocytosis influence the relative levels of amyloidogenic versus non-amyloidogenic processing [232]. More specifically, at the plasma membrane APP can be cleaved by α-secretase to release soluble sAPPα which precludes subsequent amyloidogenic processing and Aβ formation [12]. Indeed, it has been suggested that reduced α-secretase activity might be a contributing factor to AD pathogenesis [233]. The unprocessed APP molecules that avoid non-amyloidogenic processing are internalized through clathrin-mediated endocytosis [232]. These internalized APP molecules can be recycled to the cell surface or undergo amyloidogenic processing [12]. 

Studies have demonstrated that the WNT co-receptor LRP6 binds to APP and reduces the rate of endocytosis, subsequently elevating non-amyloidogenic processing by 𝛼-secretase and concurrent reduction in Aβ production [195]. Other studies suggest WNT signaling regulates APP endocytosis through lysosomal activity [196]. Specifically, WNT5a was shown to bind to APP and regulate its trafficking by decreasing APP levels in the trans-Golgi network and increase the levels of lysosomes. Normal APP trafficking was restored through inhibition of lysosomal activity [196]. This same study also found that WNT3a binds to APP and regulates retrograde APP trafficking, whereas retromer inhibition reversed this effect. 

#### 4.2.3. Regulation of APP processing by WNT Signaling

APP not processed at the cell surface by α-secretase is internalized from the plasma membrane to early endosomes [12,14]. From early endosomes it can either enter recycling endosomes to be trafficked back to the cell surface or travel back to the trans-Golgi network via retromer-mediated pathways [17]. Alternatively, early endosomes can mature into late endosomes where the increasingly acidic pH fosters beta-amyloid precursor protein cleaving enzyme (BACE1) activity and cleavage of APP to generate CTF-β, which is then cleaved by γ-secretase to generate Aβ [14]. In neurons, a large percentage of Aβ is degraded by a variety of endosomal enzymes and proteases [14]. In lieu of intraneuronal degradation, Aβ can be exocytosed to the extracellular space [14]. In AD patient brains, these various cleaved products of APP amyloidogenic processing accumulate inside neurons [19]. In particular, increased intraneuronal accumulation of CTF-β in enlarged endosomal compartments is a pathological hallmark of AD progression and thought to contribute, in part, to downstream synaptic loss [17].

As it relates to WNT signaling, the WNT ligands WNT3a and WNT5a were also shown to regulate amyloidogenic processing and Aβ via activation of canonical WNT/β-catenin and WNT/PCP signaling, respectively [197]. Activation of canonical and non-canonical WNT signaling increases non-amyloidogenic processing of APP and decreases amyloidogenic APP processing. Moreover, activation of WNT signaling decreased the Aβ42/Aβ40 ratio [198]. In this same study, activation of WNT signaling reduced BACE1 levels via β-catenin dependent transcription [198]. Mechanistically, another study showed that *Bace1* transcription was repressed by binding of Tcf4 to the promoter of Bace1 during active canonical WNT/β-catenin signaling [199]. Alternatively, inhibition of WNT signaling using ICG001 or XAV939 increased amyloidogenic processing in animal models of AD [222].

#### 4.2.4. Interaction of APP and PSEN1 with Components of the WNT Signaling Pathway 

Numerous studies have shown that APP and presenilin-1 (PSEN1) can reciprocally regulate WNT signaling activity which might impact downstream AD-related phenotypes. For example, one study demonstrated that APP binds to β-catenin in vitro and in vivo [234]. In the same study, overexpression of APP was observed to modulate the distribution of β-catenin from the nucleus to the cytoplasm and alter WNT target gene expression. Another study investigating the AICD, produced after cleavage of full-length APP, revealed that AICD inhibited WNT signaling [235]. More specifically, this study showed that AICD interacts with and activates GSK3β, enhancing its kinase activity and degradation of cytoplasmic β-catenin. As a result, AICD-mediated GSK3β activity inhibited canonical WNT/β-catenin signaling as demonstrated by repressed expression of the WNT target gene c-Myc in neural cells [235]. Further studies have suggested that modulation of phosphorylated GSK3β and nuclear β-catenin levels may contribute to AD progression through altered WNT signaling [234].

PSEN1 has also been revealed to interact with components of the WNT signaling pathway including β-catenin and GSK3β. For example, it has been shown that PSEN1 complexes with β-catenin to increase its stability in the cytoplasm and is mostly localized in the ER and Golgi [236,237]. However, β-catenin levels are significantly reduced in the brains of AD patients with *PSEN1* mutations [238]. Zhang and colleagues demonstrated that pathogenic mutations to the *PSEN1* gene reduce the ability of PSEN1 to stabilize β-catenin, and thus prevent β-catenin degradation [238]. Additionally, pathogenic *PSEN1* mutations were discovered to cause the mis-trafficking of β-catenin translocation to the nucleus when PSEN1 and β-catenin were complexed [239]. Takashima and colleagues showed that tau and GSK3β bind to residues 250–298 of PSEN1 [200]. In turn, this increased PSEN1 binding activity with GSK3β led to greater phosphorylation of tau and downstream formation of pathogenic tau aggregates [200]. In addition, it is hypothesized that mutated PSEN1 could play a regulatory role in the Akt/GSK3β apoptotic cascade. In this vein, it has been observed that inhibition of the PI3K/Akt signaling pathway leads to activation of GSK3β and apoptosis [240,241]. As such, increased activation of GSK3β mediated by mutated PSEN1 might increase the vulnerability of the cell to stressful, pro-apoptotic stimuli, such as neurotoxic Aβ peptides [242]. Together, these studies show that pathogenic mutations in *PSEN1* can regulate different components of the WNT signaling pathway to promote AD-associated pathologies.

### 4.3. Role of WNT Signaling in Modulating Tau-Related Phenotypes in AD

Several studies have shown that WNT signaling and GSK3β regulation play a role in modulating tau phosphorylation. Tau phosphorylation is mediated by a variety of kinases including GSK3β [243]. As such, it has been shown that GSK3β phosphorylates AD-associated tau residues [201,202]. Indeed, inhibition of WNT signaling induces GSK3β-mediated tau phosphorylation [209,244]. Moreover, it has been suggested that DKK1 is responsible for this WNT inhibition and subsequent elevation of tau phosphorylation [213,244]. Alternatively, activation of canonical WNT signaling has been shown to reduce pathological tau phosphorylation in APP-PS1 mice through its action on GSK3β [245]. Similarly, activation of WNT signaling can also inhibit Aβ-induced tau phosphorylation [246]. Because of the role of WNT signaling in modulating tau-related pathologies, several therapies have been explored to utilize GSK3β inhibitors to increase neuronal β-catenin levels and prevent GSK3β-mediated tau phosphorylation [209,247]. However, it should be noted that it has been demonstrated that WNT signaling regulates the inflammatory response in microglia [204]. Other experiments investigating the relationship between microglia and tau using mouse models found elevated levels of IL-1, a pro-inflammatory cytokine, exacerbated tau phosphorylation [248,249]. Thus, it could be suggested that inhibition of WNT signaling long-term through GSK3β inhibition could exacerbate pro-inflammatory responses and promote enhanced neurotoxicity.

### 4.4. WNT and Inflammatory Responses in AD

Recent investigations of WNT and microglial activation have demonstrated a dichotomy between anti- and pro-inflammatory microglia phenotypes during active WNT signaling. Studies have shown that activation of canonical WNT/β-catenin signaling inhibits microglial activation and ameliorates neuroinflammation [250,251,252]. Canonical WNT/β-catenin activation via TWS119 shifted microglia phenotype from pro- to anti-inflammatory, promoting neurological recovery following ischemic stroke in a mouse model [251]. Another study demonstrated that inhibition of canonical WNT/β-catenin signaling by AXIN2-mediated β-catenin degradation drove the transformation of microglia to an active pro-inflammatory phenotype. In turn, microglial activation and pro-inflammatory response could be reduced via the delivery of a WNT agonist [203]. These studies provide evidence for WNT-mediated regulation of protective microglia phenotypes. 

As it relates to AD, WNT signaling activity has been shown to increase microglia survival during AD. For example, one study showed that microglia survival and microgliosis was restored following WNT activation via treatment with Wnt3, LiCl, or TDZD-8 [253]. Another study revealed that the loss of neuronal LRP6 led to a significant increase in microglia activation and neuroinflammation [195]. Contrary to these findings, other studies have demonstrated WNT activation led to activation of microglia and a pro-inflammatory response evidenced by high expression of cytokines IBA1, CD68, and CD11b [254,255,256,257]. Results in another study showed that WNT signaling increased the expression of pro-inflammatory cytokines in microglia [204]. Microglia under neuroinflammatory conditions were observed to have elevated expression of β-catenin. Activation of WNT signaling by WNT3a showed increased expression of pro-inflammatory immune response genes in microglia, as well as stimulate inflammatory transformation in microglia [204]. Findings from this study suggest active WNT signaling in microglia serves to exacerbate AD-related neuroinflammation compared to other studies demonstrating WNT-associated mediation of AD pathologies. These contrasting findings bring to light a potential cell-type dependent effect of WNT signaling on microglial activation and phenotypes and subsequent AD-related pathology.

Mechanistically, numerous studies have demonstrated crosstalk between TREM2-mediated microglial activation and WNT signaling. In one such study, RNA-seq of a *Trem2* knockout in a PS2-APP AD mouse model revealed a reduction in positive regulators of canonical WNT signaling such as *Fz9*, *Sulf2*, *Bambi*, *Ptk7*, *Aspm*, and *Dkk2* [258]. In further support of this interaction, another study demonstrated that the proliferative response of activated microglia utilized both TREM2 and WNT signaling [253]. These *Trem2* knockout mice were observed to have reduced microglia proliferation and survival, but increased microglial apoptosis. Concurrently, reduced protein levels of β-catenin and inactive GSK3β were observed. Together, these data suggest that TREM2 functioned to inactivate GSK3β by phosphorylation via PI3K/Akt signaling, thus causing accumulation of β-catenin and transcription of survival genes.

### 4.5. The Role of DKK1 and LRP6 in Synaptic Loss in AD

Studies interrogating aberrant WNT signaling have revealed a potential connection to mediating synaptic dysfunction and synapse loss in AD. It has been well-established DKK1 promotes AD pathologies such as Aβ-mediated neurotoxicity and synapse loss [205,206,259]. For example, exposure to Aβ oligomers induced the expression of DKK1 and caused loss of synaptic sites whereas inhibition of DKK1 reversed Aβ-mediated synaptic loss [205]. In addition, Purro and colleagues observed that Aβ rapidly increased expression of DKK1 which functioned to disassemble synaptic components leading to synaptic loss, indicating that DKK1 is required for Aβ-mediated synaptic loss. In addition to canonical WNT signaling, DKK1 induced activation of non-canonical WNT/PCP signaling pathway which also caused Aβ-driven synapse disassembly [206,207]. This Aβ-mediated synapse loss was DKK1-dependent and required activation of the non-canonical WNT/PCP pathway [206]. The relationship between Aβ and DKK1 can be described by a positive feedback loop in which Aβ induces expression of DKK1 [207,213] which promotes synaptic loss and increased Aβ production.

Additional evidence indicates that LRP6 and WNT signaling are necessary for maintaining synaptic integrity, function, and memory. Deficiency of the WNT receptor LRP6 exhibited increased APP processing and exacerbated Aβ pathology in an amyloid mouse model [195]. This finding suggests that AD progression is accelerated by decreased LRP6-mediated WNT signaling. Another study demonstrated that APP was a co-activator of both canonical WNT/β-catenin and non-canonical WNT/PCP signaling pathways through interactions with the WNT-associated LRP6 and Vangl2 receptors [260]. In addition, LRP6 binds to the E2 domain of APP [261] preventing further cleavage by secretases and limiting Aβ deposition [195,260]. Consequently, activation of canonical WNT signaling enhanced synapse stability and reduced Aβ production, whereas activation of WNT/PCP pathway caused synaptic retraction and increased Aβ production [260]. 

Together, these studies demonstrate that WNT signaling dysfunction leads to Aβ production and associated synapse loss [260,262]. Thus, several studies have explored whether restoration of WNT signaling can ameliorate Aβ-induced synaptic loss. For example, WASP-1, a synergistic ligand of WNT3a, blocked Aβ aggregation in vitro and reduced pathological tau phosphorylation in vivo [208]. In turn, WASP-1 expression could rescue hippocampal synaptic impairments induced by AD pathologies such as Aβ oligomers. Another study investigating Aβ oligomers revealed that activation of non-canonical WNT signaling could prevent post-synaptic damage caused by these oligomers [263].

## 5. Future Trends: Improvement in Model Systems

Part of the difficulty of reconciling the role of WNT and AD have been limitations in the models used [264]. Experimental model systems and conditions vary greatly between studies proving increased difficulty in elucidating the effects of upregulation or downregulation of 𝛽-catenin stability during AD. An additional consideration in these models is the differentiation between analysis of cytoplasmic 𝛽-catenin, and nuclear 𝛽-catenin. A majority of studies report on cytoplasmic 𝛽-catenin which does not correlate with transcriptional changes by regulation from the 𝛽-catenin-TCF/LEF complex. Numerous FAD models have been employed to investigate the effects of WNT signaling during AD, however FAD only represents roughly 5% of total AD cases in humans [265]. Notably, 𝛽-catenin was only slightly reduced in SAD patients compared to controls, unlike in FAD [238]. In turn, this necessitates SAD models with limited confounding variables to investigate the extent to which WNT signaling affects AD pathology.

### Human Induced Pluripotent Stem Cell (hiPSC)-Based Approaches

Human cell models represent great platforms for genetic studies as the intricate nature of human diseases cannot be fully recapitulated by animal models. Major evolutionary differences between human and animal models contribute to the structural and biological complexity of human tissues and organs [266,267,268,269]. In turn, human induced pluripotent stem cells (hiPSCs) have emerged as a powerful resource to elucidate the underlying mechanisms of pathogenic diseases including AD. AD animal models are hindered by their complexity due to confounding variables making investigation of molecular, biochemical, and cellular phenotypes more difficult. The ex vivo culture of neuronal cells extracted from cadaveric tissues provides other modalities to studying AD, however the sources of these cells are scarce and disease phenotypes are rapidly lost after prolonged culture. As a result, hiPSCs have become an attractive model due to unlimited self-renewal and their ability to differentiate into nearly any cell type of the human body. In addition, numerous protocols have been developed to direct the differentiation of hiPSCs to a myriad of neural cell types including neurons [270,271,272], astrocytes [273,274], microglia [275], and oligodendrocytes [276]. Additionally, studies have demonstrated that FAD and SAD neural cells derived from hiPSCs exhibit disease relevant phenotypes such as elevated secreted Aβ42 levels, increased Aβ42/40 ratio, and hyperphosphorylation of tau [101,277,278,279]. More recently, CRISPR-based gene editing techniques have been applied for the generation of AD-associated disease hiPSC lines [280,281]. These isogenic lines are advantageous as they establish a defined condition and mitigate confounding variables due to age, sex, or gender identity between patient and controls to more closely study genetic modifications [282,283]. In the future, these hiPSC-based methods can be employed to resolve some of the unanswered questions as it relates to the multifaceted role of WNT signaling in AD onset and age-related progression. 

## 6. Conclusions

A link between WNT signaling and AD-related phenotypes has been well-established through epidemiological analysis, clinical studies, and animal models. Specifically, WNT signaling influences AD-associated pathologies including Aβ deposition, tau hyperphosphorylation, inflammatory response, synaptic loss, and cognitive decline. Although the cellular and molecular mechanisms by which WNT modulates such pathologies has not been precisely defined, it has been suggested that WNT signaling regulates multiple levels of the pathological hierarchy. As it relates to amyloid pathologies, WNT plays multiple roles in APP processing including regulating APP post-translational phosphorylation, altering cell surface APP levels and endocytosis, modulating APP endosomal trafficking and processing, and influencing Aβ degradation. In addition, WNT signaling regulates the kinases responsible for phosphorylating tau, mediates chronic inflammatory processes induced by microglia activation, and influences synaptic integrity. Moving forward, therapeutic advances that seek to modulate WNT signaling and its dysfunction in AD will require a more thorough understanding of the multiple effects of WNT at these various pathological levels. 

## Figures and Tables

**Table 1:** Summary of the effects of components of the canonical and non-canonical WNT signaling pathways on AD-related processes and phenotypes. Arrows (↑, ↓) in the table indicate an increase or decrease in the effect and AD-associated phenotype, respectively.

Effect	AD-Associated Phenotype	WNT Signaling Pathway	WNT Component	Reference
↑ APP phosphorylation	↑ Aβ	Canonical	GSK3β	[190,191]
↑ APP phosphorylation	↑ Aβ	Non-canonical (WNT/PCP)	JNK	[192,193,194]
↓ APP endocytosis	↓ Aβ	Canonical	LRP6	[195]
↓ APP endocytosis	↓ Aβ	Non-canonical	WNT5a	[196]
↓ APP endocytosis	↓ Aβ	Canonical	WNT3a	[196]
↑ non-amyloidogenic processing	↓ Aβ	Canonical	WNT3a	[197]
↑ non-amyloidogenic processing	↓ Aβ	Non-canonical (WNT/PCP)	WNT5a	[197]
↓ BACE1 expression	↓ Aβ	Canonical	TCF4	[198,199]
↑ PSEN1 binding activity	↑ p-tau	Canonical	GSK3β	[200]
↑ GSK3β phosphorylation activity	↑ p-tau	Canonical	GSK3β	[201,202]
↑ β-catenin degradation	↑ pro-inflammatory microglia	Canonical	AXIN2	[203]
↓ neuronal LRP6	↑ neuroinflammation	Canonical	LRP6	[195]
↑ pro-inflammatory gene expression	↑ neuroinflammation	Canonical	WNT3a	[204]
↑ DKK1 expression	↑ synapse loss and Aβ	Canonical	DKK1	[205]
↑ DKK1 expression	↑ synapse loss	Non-canonical (WNT/PCP)	DKK1	[206,207]
↓ LRP6 expression	↑ synapse loss and Aβ	Canonical	LRP6	[195]
↓ Aβ aggregation	↓ p-tau and synapse loss	Canonical	WASP-1	[208]

## Data Availability

Not applicable.
